# IUC26658-92 Efficacy Patterns of First-Line Immune Checkpoint Inhibitor Combinations in Metastatic Clear Cell Renal Cell Carcinoma: A Systematic Review and Meta-Analysis Evaluating Ethnic Variations

**DOI:** 10.1093/oncolo/oyag286.017

**Published:** 2026-08-21

**Authors:** D Khanna, T Dey, E Blanchard-Cavagis, B Gonsalves, A Benedict, M Job, D Omoregie, S Basu, O Benny, A Maniam, G L Banna, R Kanesvaran, Y Urun, K Jatwani, M N Gandur-Quiroga, M Anwar, J K Tan, S E Rebuzzi, R D Frazer, A Ghose

**Affiliations:** University of Buckingham Medical School, Crewe - United Kingdom; Sanjay Gandhi Post Graduate Institute of Medical Sciences, Lucknow, India - India; Université Paris Cité, Paris, France - France; Kings College London GKT School of Medical Education, London - United Kingdom; Barts and The London School of Medicine and Dentistry, London - United Kingdom; Keele University School of Medicine, Staffordshire - United Kingdom; Keele University School of Medicine, Staffordshire - United Kingdom; University College London, London, UK - United Kingdom; Keele University School of Medicine, Staffordshire - United Kingdom; Department of Medical Oncology, Portsmouth Hospitals University NHS Trust, Portsmouth - United Kingdom; Department of Medical Oncology, Portsmouth Hospitals University NHS Trust, Portsmouth - United Kingdom; National Cancer Centre Singapore - Singapore; Ankara University Cancer Institute, Ankara - Turkey; George Washington Cancer Centre, Washington DC - United States; Institute of Oncology Angel H Roffo, Buenos Aires - Argentina; Velindre Cancer Centre Cardiff - United Kingdom; University of Manchester - United Kingdom; Velindre Cancer Centre, Cardiff - United Kingdom; Velindre Cancer Centre, Cardiff - United Kingdom; Velindre Cancer Centre, Cardiff - United Kingdom

## Abstract

**Background:**

First-line immune checkpoint inhibitor (ICI)-based combinations have transformed metastatic renal cell carcinoma (mRCC) outcomes. However, the influence of patient ethnicity remains poorly characterized due to historical underreporting in major clinical trials. This study performed an ethnicity-based benchmarking analysis of therapeutic outcomes across standard-of-care ICI combination regimens.

**Methods:**

A systematic search of PubMed, Embase, and Cochrane databases was conducted to identify clinical studies evaluating adult patients with clear-cell mRCC treated with four European Society for Medical Oncology (ESMO) guideline-recommended regimens: Ipilimumab+Nivolumab, Nivolumab+Cabozantinib, Pembrolizumab+Axitinib, or Pembrolizumab+Lenvatinib. A random-effects meta-analysis calculated and compared pooled Objective Response Rates (ORR) for the Ipilimumab+Nivolumab cohort, stratified by Asian versus Caucasian ethnicity. Progression-Free Survival (PFS) was synthesized descriptively due to reporting heterogeneity.

**Results:**

Meta-analysis for the Ipilimumab/Nivolumab regimen encompassed data from a total pooling of 3,489 patients. The quantitative synthesis revealed a pooled ORR of 44% (95% CI: 39%–49%) within the Asian cohorts, compared to a pooled ORR of 40% (95% CI: 35%–46%) in the Caucasian cohorts. Statistical testing demonstrated that this minor variance in response rates between the two ethnic subgroups was not significant (𝑋^2^  =  1.08$, p  =  0.298). High levels of heterogeneity were observed within both arms (*I*^*2*^  =  73.8%) for Asians (*I*^*2*^  =  85.1%) for Caucasians. In alignment with the ORR findings, the descriptive analysis of median PFS demonstrated substantial overlap between the Asian and Caucasian groups, revealing no clear ethnicity-driven disparities in disease control. Crucially, data evaluating the other three ICI-tyrosine kinase inhibitor (TKI) combinations were sparse. Furthermore, the reporting of outcomes for Black and Mixed-ethnicity populations was profoundly deficient across the literature, which precluded any meaningful subgroup analyses for these cohorts.

**Conclusions:**

The Ipilimumab+Nivolumab regimen demonstrates consistent clinical efficacy across Asian and Caucasian populations, with no statistically significant or clinically meaningful divergence in ORR or PFS. However, the global body of evidence in mRCC remains severely limited by high statistical heterogeneity and a stark underrepresentation of Black and Mixed-ethnicity patients. Standardized and ethnically inclusive demographic reporting in future oncology trials is vital to ensure equitable global application of these therapies.

